# Assessment of Clinical Reasoning and Diagnostic Thinking among Dental Students

**DOI:** 10.1155/2022/1085326

**Published:** 2022-09-26

**Authors:** Fatemeh Owlia, Fatemeh Keshmiri, Maryam Kazemipoor, Fahimeh Rashidi Maybodi

**Affiliations:** ^1^Department of Oral and Maxillofacial Medicine, School of Dentistry, Shahid Sadoughi University of Medical Sciences, Yazd, Iran; ^2^Medical Education Department, Educational Development Center, Shahid Sadoughi University of Medical Sciences, Yazd, Iran; ^3^Faculty of Public Health, Shahid Sadoughi University of Medical Sciences, Yazd, Iran; ^4^Department of Endodontics, School of Dentistry, Shahid Sadoughi University of Medical Sciences, Yazd, Iran; ^5^Department of Periodontics, School of Dentistry, Shahid Sadoughi University of Medical Sciences, Yazd, Iran

## Abstract

**Introduction:**

This study aimed to investigate dental students' clinical reasoning and diagnostic thinking ability by key feature test and “diagnostic thinking inventory” questionnaire.

**Methods:**

The present study was a descriptive cross-sectional study. The participants consisted of 61 senior dental students. Clinical reasoning and diagnostic thinking were assessed by key feature tests and the “diagnostic thinking inventory” “DTI” questionnaire, respectively. The “diagnostic thinking inventory” was developed by Bordage et al. in France and consisted of 41 questions on a 6-point Likert scale. The satisfaction of students was assessed through a 10-item questionnaire. Data were analyzed using SPSS 19 with descriptive tests (mean, SD, and percentage), student independent *T*-test, and Pearson correlation. The significance level was determined at *p* < 0.05.

**Results:**

The mean scores of the key feature test were 56.55 ± 7.80. Diagnostic thinking scores of learners were reported in diagnostic thinking 136.47 ± 16.45, flexibility in thinking 72.22 ± 11.15, and structure of memory 64.24 ± 7.84. The difference in students' scores in flexibility in thinking was significantly higher among male students than female students. (*p*-value = 0.04). The students' satisfaction scores were 3.53 ± 0.52, which showed relative satisfaction.

**Conclusion:**

The participants' clinical reasoning and diagnostic thinking skills were reported at a low level. This issue emphasizes the need for training to enhance diagnostic thinking and clinical reasoning in dental education. Formative evaluation and reform of the educational programs of this course should be considered.

## 1. Introduction

Clinical reasoning is considered one of the most critical skills required for health personnel [1]. Clinical reasoning is a logical thinking process that guides health providers to take purposeful steps in diagnosis and treatment. This process exists in all stages of dealing with the patient, from the initial stage of getting the history of the disease to the patient management and follow-up [2]. Clinical reasoning as a clinical competency is considered essential and has been introduced as one of the essential skills for conducting professional responsibility and responding to patients [3]. Clinical reasoning consists of four components: data collection, clinical hypothesis formation, hypothesis testing, and clinical decision-making and problem solving. Educational methods such as problem-based learning, team-based learning, and case-based learning are recommended to develop the reasoning skills of medical students [4]. Insufficient attention to teaching clinical reasoning skills and related concepts increases the missed diagnosis as the main cause of medical errors [5]. Therefore, teaching clinical reasoning to students of medical sciences is essential. Most medical graduates do not have the right clinical reasoning skills to diagnose and perform effective and safe interventions for patients [6]. This topic highlights the importance of teaching critical thinking skills and clinical reasoning.

Educational systems aim to switch the memorization approach to developing learners' reasoning and problem-solving skills [7]. Clinical reasoning training provides students with the opportunity to achieve critical thinking, clinical decision-making, and learning in three areas, cognitive, affective, and psychomotor, to prepare them for their professional role in society [8]. In dentistry, decision-making and reasoning skills are vital in oral and maxillofacial medicine. The main activities of this field are the ability to diagnose and treat oral mucosal lesions, salivary gland diseases, and temporomandibular joint and dental management of patients with systemic diseases. Early diagnosis of oral lesions is essential because many of them could be the first sign of undiagnosed systemic disease or lead to a malignant lesion with a late diagnosis of a premalignant lesion. Currently, the dentistry curriculum mainly focuses on the development of procedural skills, although there is a need to change the training processes and evaluate clinical reasoning skills in the school of dentistry.

The key-feature (KF) test has introduced a subset of clinical reasoning tests. KF is a scenario-based clinical exam that solves the clinical problem through several questions about necessary actions or clinical decisions [9]. In this test, a short scenario with little information is provided to the examiner, and he/she detects critical information by selecting the choices. The value of responding to different options in the KF test varies and could be changed according to their values in the patient's diagnosis and treatment. The KF test assumes that not all information is of equal value, but key points are more critical in solving the problem than other symptoms. A mistake in identifying them will cause the failure to solve the problem correctly. This test focuses on more valuable points of diagnosis and treatment than others.

The advantages of this test include the ability to evaluate clinical decision making, the possibility of broader coverage of clinical cases compared to other reasoning tests, and the design of various forms of answers. High reliability and the possibility of a more structured and focused evaluation of clinical cases were introduced as the benefits of the test [10]. According to studies, the KF test is a practical test for measuring the clinical reasoning skills of medical students, and its score is closer to the scores of the objective structure clinical examination (OSCE) test that is provided a simulated situation for students' assessment [11].

**Figure 1:**
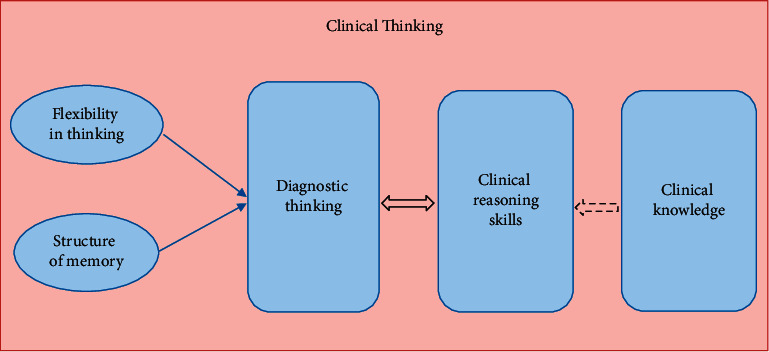
Conceptual framework of the study.

Other tools were introduced to assess clinical reasoning skills. One is the diagnostic thinking inventory (DTI) [12]. DTI is a tool that measures the flexibility in thinking and structure of knowledge in memory independently of content and can evaluate different degrees of medical skills [13]. DTI is a suitable tool for comparative assessment of clinical reasoning ability based on self-expression among students with different curricula. Diagnostic thinking differs from critical thinking, and both are used to assess clinical reasoning [14]. Hamzeh et al. compared clinical competency using diagnostic thinking inexperienced and novice physiotherapists. Their results showed that the DTI instrument had sufficient validity and reliability to assess clinical diagnostic reasoning [15]. A few studies were found about assessing clinical reasoning and diagnostic thinking among dental students. The education and assessment of clinical reasoning were not considered in the formal curriculum of dentistry school. In the first step, the state of clinical reasoning of the students was measured. If necessary, educational interventions should be made in subsequent studies. We hypothesized that the diagnostic thinking abilities of dental students correlated with their clinical reasoning. We have shown our conceptual framework in Figure 1. This study determined dental students' clinical reasoning and diagnostic thinking abilities through key feature tests and DTI questionnaires.

## 2. Method

**Figure 2:**
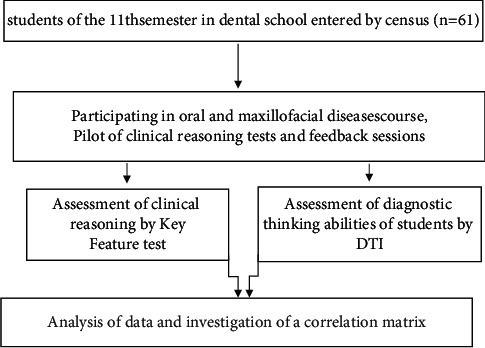
Flow chart of the study steps.

The present study was a descriptive cross-sectional study (Figure 2).

### 2.1. Participants

The study population comprised 61 students in the 11^th^ semester in a dental school affiliated with Shahid Sadoughi University of Medical Sciences that were entered by the census.

### 2.2. Instruments

The DTI was developed by Bordage et al. in France and consisted of 41 questions on a 6-point Likert scale [16]. Of which, 21 are memory structure category, and 20 are flexibility in thinking category. Based on this questionnaire, individuals use a tool to evaluate themselves in different situations, and DTI has an acceptable validity (*α* = 0.83) that can distinguish between mastery and novice diagnosis [16]. This questionnaire was psychometric in the Soltani-Arabshahi study in the Iran University of Medical Sciences context [12]. The satisfaction questionnaire consisted of 10 questions. (Cronbach's alpha = 0.85). The scoring of the questionnaire was on a 5-point Likert scale. We have also used the KF test to assess dental students' clinical reasoning.

To design the KF test questions, the exam blueprint was first designed by an expert panel based on the dental curriculum. Based on Nayer et al. study [11], to achieve the desired reliability in this process, 17 questions were designed and reviewed after design (Table 1). The questions were developed based on standard cases of oral and maxillofacial diseases by the group of oral and maxillofacial specialists.

In order to inform students about the KF test, a pilot study was conducted to familiarize them with reasoning questions at the beginning of the semester. Students spend a one-month course on oral and maxillofacial diseases. All participants participated in the study with ethical considerations, provided they wanted to participate. Key feature test scores were calculated from zero to 100. In addition to the instant feedback included in the questions, a face-to-face feedback session for students was scheduled and conducted the next day after the test. Moreover, the students completed the DTI questionnaire.

### 2.3. Data Analysis

Data were analyzed using SPSS 19 with descriptive tests (mean, SD, and percentage), student independent *T*-test, and Pearson correlation. The significance level was considered *p* < 0.05.

### 2.4. Ethical Consideration

Eligible participants were informed about the aim of the study, the method, and the confidentiality of their responses by e-mail. In this study, informed consent was obtained from the participants. This study was approved by the Ethics Committee Research at Shahid Sadoughi University of Medical Sciences (IR.SSU.REC.1399.302).

## 3. Results

### 3.1. Participants

Sixty-one dental students, including (36 (59%) men and 25 (41%) women) with a mean age of 23 ± 2.76, were enrolled in the present study. DTI scores of students were shown in Tables 2 and 3.

There was no significant difference in the diagnostic thinking scores between males and females (*p*-value = 0.11). This significant difference was not reported in the domain of memory structure (*p*-value = 0.66). However, the difference in students' scores in the flexibility in thinking domain was significantly higher among male students than female students. (*p*-value = 0.04) (Table 4).

The Pearson correlation results showed no significant correlation between students' diagnostic thinking scores and their clinical reasoning scores in the key feature test (*r* = 0.16, *p*-value = 0.19). There was no significant relationship between learners' key feature test scores with the DTI test (*r* = 0.16, *p*-value = 0.19) and DTI's domains including diagnostic thinking flexibility (*r* = 0.15, *p*-value = 0.24) and memory structure (*r* = 0.14, *p*-value = 0.32).

The students' satisfaction scores were 3.53 ± 0.52, which showed relative satisfaction with the reasoning test during the oral and maxillofacial disease. The lowest score was 2.27, and the highest score was 4.90. There was no significant difference between the satisfaction levels of male and female participants (*p*-value = 0.12).

The mean scores of the key feature test are shown in Table 5. No significant difference was reported between clinical reasoning scores of key feature test by students' gender (*p*-value = 0.19). DTI scores of students in different domains by gender are shown in Table 6. A correlation matrix of students' diagnostic thinking and clinical reasoning scores are shown in Table 7.

## 4. Discussion

In this study, a key feature test and DTI questionnaire were used to assess the clinical reasoning of senior dental students. In each clinical case, some critical points were embedded that should be considered in clinical independent decision making and in combination with other factors. The KF is a test based on an individual's clinical reasoning that solves the patient's problem step by step [17].

In the present study, the scores of the KF test were at a low level, and no difference was observed between the two genders. It is worthy of being mentioned that the achievement of the clinical, educational approach should be applied in dental schools. In midwifery school, the students experienced an experimental cycle in education. They encountered key feature examination with pretraining seminars that would demonstrate better achievements in education than usual tests. Decision-making ability and problem-solving skills could be improved by clinical reasoning teaching among midwifery learners [17]. The present study showed moderate satisfaction, which was consistent with the results of the branch study [18].

Due to the shortage of a proper model of conducting exams in the approved curriculum of dentistry in Iran, clinical reasoning and problem-solving skills have been ignored. All medical science educational systems are responsible for shifting medical learners from memorizing content to reasoning and creative problem solving. In the present study, similar to the study of Soltani-Arabshahi et al., the Iranian version of the DTI questionnaire was used to assess the clinical reasoning of dental students. The results of the study by Arabshahi et al. showed that the participants' mean score of clinical reasoning was moderate, and diagnostic thinking was not significantly different between residents and interns in terms of components of memory structure and flexibility in thinking. The obtained results showed that the DTI scores of senior dental students were less than that among medical learners in the Arabshahi study. Regarding flexibility in thinking, the results of both studies were close to each other, but the score of memory structure was higher among medical students in the Arabshahi study [12]. The result may achieve the investigated dental school which focused on improving the procedural skills of learners more than the thinking abilities such as critical thinking, diagnostic thinking, and reasoning skills.

It should be noted that DTI is a test based on participants' self-expression, and its subjective nature can affect the results. Tajvidi and Hanjani showed a direct and positive relationship between clinical reasoning and critical thinking in nurses [19]. In 2015, Sajadi compared the ability of dental students to answer multiple-choice questions and clinical reasoning and calculated their relationship to academic achievement. They concluded that despite good academic achievement, students' ability to answer the clinical reasoning test was low, and they gained lower scores than on multiple-choice tests [20].

In the present study, there was no significant difference in diagnostic thinking between males and females, similar to Gehler et al. No significant relationship was reported in the domain of flexibility in thinking. In the study of Gehler et al. there was a significant difference between the two genders regarding flexibility in thinking, and the scores of men were better than those of women [21]. This result was inconsistent with Tajvidi and Hanjani study, in which females scored better on clinical reasoning. Diagnostic thinking was poor among dental students in the present study, which was consistent with the results of the study by Tajvidi and Hanjani. Also, Tajvidi and Hanjani reported a moderate level of clinical reasoning ability in nursing students and nurses [19]. Their participants' scores were higher than students in the present study. In their study, the reasoning ability of nurses was measured, while in the present study, the reasoning ability of senior dental students who did not have experience in clinical practice and service delivery as a healthcare provider. In addition, the differences in curriculum and clinical education and participants' experience may affect the results. It seems that administering clinical reasoning tests in medical education systems as a valid examination would be necessary for developing countries. Therefore, it is recommended to conduct further studies to assess the influence of the experience factor on the development of reasoning skills among dental students and dentists.

Despite obtaining the mean score on the KF test, student's satisfaction with the test was moderate. The existence of structured responses is one of the reasons for increasing learners' satisfaction with this type of test. Most were satisfied with the statement, “This test allows me to understand my strengths and weaknesses in the process of diagnosis and management of oral lesions,” so it can be said that the use of reasoning tests by providing immediate feedback and objective structured answers can be effective and provide better learning opportunities for students. In addition, in the present study, a face-to-face feedback session was held to improve the test's educational impact. Due to the low level of clinical reasoning among the participants of the present study, similar to other studies in the Iranian context, the planning for teaching and evaluating clinical reasoning in a codified way in different departments of dental schools is necessary.

One of the limitations of the present study was the small number of participants in the investigated dental school. In addition, the complexity of understanding the questions of the DTI took much time for the test takers to complete the questionnaire, which can effectively answer the final questions.

## 5. Conclusion

The present results showed that dental students' clinical reasoning and diagnostic thinking skills were reasonably low. Our results showed no significant correlation between students' diagnostic thinking scores and clinical reasoning scores in the key feature test. Training to enhance diagnostic thinking and reasoning capabilities in dental education is critical. In order to improve this skill in dental students, there is a need to reform the educational programs of this course and assessment plan in the dental school. The use of clinical reasoning tests as a formative examination in different educational stages is recommended.

## Figures and Tables

**Table 1:** The detail of key feature examination.

Number of questions	Field of evaluation	Disease	The goal of evaluation
1	Dental management	Seizure	(i) Students must know the dental management of convulsive patient
4	(i) Diagnosis (ii) Laboratory tests (2 cases) (iii) Data collection	Endocrinal disorders	(i) Students must diagnose the oral signs and symptoms of Addison disease and prescribe the laboratory tests (ii) Students should prescribe the laboratory tests for hyperthyroid patients before dental management
1	Pretreatment consideration	IV drug users	(i) Students must know pretreatment consideration of IV drug users
2	On treatment consideration	Kidney	(i) Students must know pre-extraction consideration of kidney transplant patients (ii) Students must know pretreatment consideration of hemodialysis patients
4	(i) Pretreatment consideration (ii) Dental management (2 cases) (iii) Diagnosis	Gastrointestinal	(i) Students must know pre-extraction consideration of inflammatory bowel disease patients (ii) Students must consider dental management of patients with a history of gastrectomy (iii) Students must diagnose the oral signs and symptoms of Crohn disease
1	(i) Diagnosis	AIDS	(i) Students must diagnose the oral signs and symptoms of AIDS
1	(i) Diagnosis	Leukemia	(i) Students must diagnose the oral signs and symptoms of leukemia
1	Pretreatment consideration	Anemia	(i) Students must know pretreatment consideration of anemic patients
1	Pretreatment consideration	Orofacial pain	(i) Students must diagnose the symptoms of orofacial pain
1	Pretreatment consideration	Hepatitis	(i) Student must know pre-extraction consideration of hepatitis

**Table 2:** Frequency of DTI score levels of students.

	Number (%)	Score of DTI
Very poor	54 (88.5)	<150
Poor	2 (3.3)	150–155
Moderate	2 (3.3)	156–160
Good	1 (1.6)	161–165
Very good	0 (0)	166–170
Excellent	2 (3.3)	171–246

**Table 3:** Diagnostic thinking scores of students in different domains.

	Mean ± std. Deviation	Maximum	Minimum
Diagnostic thinking	136.47 ± 16.45	175.00	93.00
Flexibility in thinking	72.22 ± 11.15	93.00	45.00
Structure of memory	64.24 ± 7.84	84.00	43.00

**Table 4:** Diagnostic thinking scores of students by gender.

	Gender	Mean ± std. Deviation	*p*-value
Diagnostic thinking	Male	139.28 ± 17.24	0.11
	Female	132.44 ± 14.64	

Flexibility in thinking	Male	74.66 ± 10.55	0.04
	Female	68.72 ± 11.26	

Structure of memory	Male	64.61 ± 8.42	0.66
	Female	63.72 ± 7.06	

**Table 5:** Clinical reasoning scores of students from the KF test.

		Mean ± std. Deviation	Maximum	Minimum
Clinical reasoning score		56.55 ± 7.80	70.00	36.71

Gender	Male	57.63 ± 7.23	70.00	42.71
	Female	55.00 ± 8.48	69.88	36.71

**Table 6:** DTI scores of students in different domains by gender.

	Gender	Mean ± std. Deviation	*p*-value
Diagnostic thinking	Male	139.28 ± 17.24	0.11
	Female	132.44 ± 14.64	

Flexibility in thinking	Male	74.66 ± 10.55	0.04
	Female	68.72 ± 11.26	

Structure of memory	Male	64.61 ± 8.42	0.66
	Female	63.72 ± 7.06	

**Table 7:** A correlation matrix of students' clinical reasoning scores and their diagnostic thinking and domains.

		DTI	Flexibility domain	Structure domain
	*N*	61	61	61
Clinical reasoning scores	Pearson correlation	0.168	0.151	0.137
	Sig. (2-Tailed)	0.196	0.244	0.294

^*∗∗*^. Correlation is significant at the 0.01 level (2-tailed).				

## Data Availability

The datasets used and/or analyzed during the current study are available from the corresponding author on reasonable request. Data were not public generality due to the confidentiality of data.
